# Advancing health research through research governance

**DOI:** 10.1136/bmj.k2484

**Published:** 2018-07-16

**Authors:** Luis Alejandro Salicrup, Luis Gabriel Cuervo, Rodolfo Cano Jiménez, Nelly Salgado de Snyder, Francisco Becerra-Posada

**Affiliations:** 1 Pan American Health Organization/World Health Organization, Washington, DC, USA; 2 Center for Global Health, National Cancer Institute, National Institutes of Health, Rockville, MD, USA; 3 Department of Health Systems and Services, Pan American Health Organization/World Health Organization, Washington, DC, USA; 4 Comisión Coordinadora de Institutos Nacionales de Salud y Hospitales de Alta Especialidad, Secretaría de Salud, México, Mexico; 5 Sistema Nacional de Investigadores Nivel III, Programa de Salud Global/Centro de Investigación en Sistemas de Salud, Instituto Nacional de Salud Pública, Cuernavaca, Morelos, Mexico

## Abstract

Good governance practices are crucial for advancing research for health in LAC countries, argue **Luis Alejandro Salicrup and colleagues**

Countries need sustainable national health research systems to maximise the benefits of health research. Importantly, these systems should be guided by national research agendas relevant to a country’s health needs to improve the effectiveness and efficiency of the health system when responding to health priorities.^1^ National health research systems may be seen as primarily assisting ministries of health, but the insights they generate can inform government sectors beyond health by providing the local insight needed to tackle inequities and social injustice, especially among the most vulnerable communities.^1^ In Latin America and the Caribbean (LAC), better organised health research systems will enhance the ability of the countries’ health systems to produce evidence informed policy, develop health programmes, and deliver preventive and treatment services.

Health research systems consist of the different institutions that support national health systems and tackle public health challenges through planning, coordinating, monitoring, and managing health research resources and activities. An important role for health research systems is integrating national context with research knowledge, the local determinants of health, as well as the authorities and stakeholders that deliver healthcare and public health. The network of institutions that make up a national health research system improve public health by translating research knowledge into better policy, processes, and administrative structures.

In 2009, all member states of the Pan American Health Organization/World Health Organization (PAHO/WHO) approved its policy on research for health.^2^ An important aim of the policy is to ensure that national health research systems have appropriate governance structures in place to enable them to function effectively. The structures that the policy aims to strengthen are those that administer and supervise how research is managed and financed, how research participants are protected, and how accountability is ensured.^2^ Governance related to research for health must also guarantee that national health research systems strengthen economic and social indicators^1^ ^3^ and support countries in fulfilling their commitments to regional and global policies and mandates.^1^ ^2^

Health research governance is a crucial component of any national health research system, guiding the roles and actions of the different individuals, organisations, and sectors involved in health research by allocating responsibilities and resources, including funding. In settings with established research governance initiatives, health systems and services have benefited from enhanced efficiency and effectiveness, including increased competitiveness.^3^ For example, health research governance practices implemented in the United States, the United Kingdom, and some European Union countries have prompted the development of innovative medicines and health technologies, as well as the ability to respond systematically to the different health challenges affecting those countries.^3^ ^4^

## Health research governance in LAC countries

A decade ago, only a handful of LAC countries had a policy or programme on research governance for health in place.^5^ Now, however, a substantial number across the region have established or have started to advance research policy initiatives and build the necessary workforce. These include Antigua and Barbuda, Argentina, Bahamas, Bermuda, Brazil, Cayman Islands, Chile, Colombia, Costa Rica, Dominican Republic, El Salvador, Guyana, Honduras, Mexico, Panama, Paraguay, Peru, St Kitts, Surinam, Uruguay, and various English speaking Caribbean countries.^6^ As countries have developed policies and progressed at different rates, the importance of alignment and standardisation of key components of their research policies has become pressing. Key elements include the need to use research evidence to deal with the challenges presented by chronic diseases, as well as the ongoing challenges posed by infectious diseases.

From our perspective, three persistent major gaps limit the role of research governance in LAC countries: the lack of effective coordination among government departments overseeing research quality; the lack of adequate funding aligned with national research priorities; and gaps in research capacity.^6^ Nearly 10 years after adopting the policy for research on health, LAC countries still need to improve health research governance to tackle their public health needs. However, successful initiatives and programmes in LAC countries provide learning opportunities.

## Current gaps in funding and coordination of health research

Although LAC countries have a common historical and cultural past, they are highly diverse in many aspects—for example, language, resources, gross domestic product, and research infrastructure. On average, LAC countries invest 0.6% of their gross domestic product in research and development, far less than the 2-3% invested by the wealthiest nations, such as the US, Germany, and Japan. Brazil spends 1.15%, Chile 0.34%, Argentina 0.6%, and Mexico 0.56%.^7^

Mexico’s Sectoral Fund for Health and Social Security (FOSISS) is an example of a sustainable research funding model in a LAC country (box 1). Chile and Uruguay have implemented similar approaches to distributing sector funds, convening an annual call for research proposals; however, funding for health research remains limited. Argentina has increased funding for biomedical research since it established its federal Ministry of Science and Technology in 2007. Under guidance from the federal Ministry of Health, the 23 provincial ministries of health and that of the Autonomous City of Buenos Aires recently held a meeting aimed at improving coordination and sharing the benefits of research agendas and specific research funding. As a result, the national authorities hope to enhance coordination of health research priorities and funding.^10^

Box 1 **FOSISS: example of a successful sustainable research funding system**

In Mexico, the National Council of Science and Technology (Consejo Nacional de Ciencia y Tecnología ( CONACYT)) is the leading government agency charged with formulating policies, programmes, and practices to promote and strengthen scientific research and innovation. In 2002, CONACYT established the sector funds (fondos sectoriales) as a way to integrate research into all government sectors. The Sectoral Fund for Health and Social Security (Fondo Sectorial de Investigación en Salud y Seguridad Social (FOSISS)) specifically supports research in those two sectors.^8^

All sector funds operate with federal secretariats/ministries and other agencies to allocate their federal research money, which CONACYT then matches and manages through a special trust for each fund. Thus, allocating funds from various sectors, CONACYT provides finance for projects that tackle the country’s needs.

FOSISS is an example of a system that funds health research in a sustainable way. It provides financial support to projects responding to critical demands and priorities of the national health system, as set out in the country’s Health Sector Programme (Programa Sectorial de Salud*)*.^8^ Its mission is to direct the country’s health research policy and priorities, with guidance from the national health authorities (Secretariat of Health (Secretaría de Salud)) and input from the other two Mexican health agencies involved in healthcare, the Mexican Social Security Institute (Instituto Mexicano del Seguro Social) and the Institute for Social Security and Services for State Workers (Instituto de Seguridad y Servicios Sociales de los Trabajadores del Estado).

FOSISS is an effective tool to tackle major obstacles generally associated with health research governance. It has improved understanding of the benefits of research, brought adequate funding, dealt with capacity gaps, and improved integration between research for health and pressing social issues, thus tackling major public health needs.^9^ Moreover, the Mexican experience with handling the sector fund and its alignment with research for health priorities has improved both transparency and accountability.

The policy approach used with FOSISS could easily be replicated or adapted in other LAC countries and beyond.

## Coordinating research governance in LAC countries

All countries need sustainable research systems to improve the health and welfare of their populations, reduce inequalities and social injustices, and promote economic and social prosperity. In LAC countries, the governance of health research remains uneven. The lack of coordination among relevant stakeholders, such as the ministries responsible for health, science and technology, education, and the economy, is a limiting factor hindering efforts to strengthen governance in several countries. For public health, this lack of coordination among stakeholders could affect the adequate distribution of available funding to support priority needs and challenges.

Paraguay is an example of where there is a lack of adequate coordination among stakeholders regarding research policies and funding programmes. Paraguay developed its national health research agenda with stakeholders, but CONACYT, the country’s National Council of Science and Technology, is the main funding agency and operates independently. CONACYT continues to fund research, but not necessarily in line with the priorities identified in the national health research agenda, which defeats the purpose of having one.^11^

More positively, the global Council on Health Research Development has spearheaded valuable efforts in conjunction with PAHO and national authorities to enhance both dialogue and coordination among the different agencies involved in health research governance in LAC countries (box 2).

Box 2 **Role of international organisations convening cross-sector dialogues among key stakeholders in health research in LAC countries**

The Council on Health Research Development, the Ministry of Health (Ministério da Saúde) of Brazil, and PAHO together hosted a discussion between the health and research sectors at a 2006 meeting in Guatemala.^12^ They aimed to tackle issues related to research governance, with the goal of increasing the impact of research on health and equity in Latin America. This was followed by the first Latin American Conference on Research and Innovation for Health, held in 2008 in Rio de Janeiro, Brazil, and organised by the Ministry of Health of Brazil, the Council on Health Research Development, the Global Forum for Health Research, and PAHO. Among other objectives, the conference focused on improving regional cooperation aimed at tackling and solving common problems. Discussions centred on the need to develop and strengthen national health research systems in Latin America, enhance the coordination among relevant stakeholders (eg ministries of health and ministries or councils of science and technology) and analyse different ways of financing biomedical and health research as well as the workforce required.^12^

Two other regional meetings were held in Cuba in 2009 and Panama in 2011.^12^ They provided a venue for learning about the characteristics of differing national health research systems in LAC countries, with input from delegates who completed a standardised form describing their respective system in the 23 countries they represented.

All three meetings^12^ provided a space to build trust and professional relationships among delegates from the two main national stakeholders, the ministries of health and of science and technology (or its equivalent). A key lesson learnt was that these conferences strengthened cross-sector collaboration in research governance and national health research systems in several LAC countries. For example, Mexico’s Secretariat of Health hosted Paraguayan and Costa Rican delegations for document sharing and visiting institutions, so they could understand how the sector fund and its governance worked.^12^

However, a lack of sustained funding has meant that these conferences have not been held regularly, and therefore a lack of sustained coordination among stakeholders remains a limiting factor hindering research governance in LAC countries.

## Building research capacity

Throughout the 1990s, LAC countries implemented a series of health sector reforms in which the goal was to increase equity, effectiveness, quality, efficiency, sustainability, and social participation. Although these reforms have had some positive outcomes in reducing inequities in access to health services and improving resource allocation, overall the reforms have not been successful in achieving the proposed goals. Reasons for this may be that research linked to public health needs and challenges was generally ignored and there is a persistent gap in skills and capacity in the research workforce of many LAC countries.^13^

Some LAC countries, particularly those in Central America and the Caribbean, have both an inadequate distribution and a shortage of healthcare providers. These problems are exacerbated by deficits in the skills required to advance and sustain research. Moreover, the lack of success in implementing some health system reforms has been linked to the failure to strengthen policy, planning, and management of the health workforce.^14^ Training scholarships are an important means of increasing research capacity in the region (box 3).

Box 3 **PAHO-OAS scholarships: for strengthening workforce capacity in research for health in LAC countries**

One mechanism to tackle the staffing gap in LAC countries is the scholarship programme on research for health coordinated by PAHO and the Organization of American States (OAS). In 2014, OAS and PAHO signed and implemented a joint agreement expanding the OAS scholarship programme to cover health related graduate studies. For 60 years, this programme has been providing scholarships to students from member states to complete masters and doctorates in diverse fields, ranging from engineering to agronomy to science and technology. The aim of the PAHO-OAS programme is to train a professional workforce from different fields in methodologies on research for health to enhance national health systems in the Americas, thus fostering the connection between research and public health. The two other main partners in the programme are organisations from Brazil and Mexico, through Brazil’s Coimbra Group of Brazilian Universities (Grupo Coimbra de Universidades Brasileiras) and Mexico’s National Council of Science and Technology and the Mexican Agency of International Cooperation for Development (Agencia Mexicana de Cooperación Internacional para el Desarrollo). The agreement emphasises development of skills and competencies to promote the production and use of research for health as a tool for informing healthcare policies and prevention, increasing universal healthcare and services, and strengthening national health systems.^15^

Between 2014 and 2017, 683 PAHO-OAS scholarships were awarded to professionals from 27 PAHO member states. Women constituted 63% of the beneficiaries of these scholarships.^16^ Despite the large number of scholarships awarded, inequity remains in terms of the beneficiaries. In particular, fewer applicants are from countries in Central America and the Caribbean, where the need to strengthen health systems through research is important. Between 2014 and 2017, less than 2% of applicants came from the most vulnerable ethnicities in LAC countries—for example, indigenous peoples and Afro descendants. This gap is important because these groups are particularly affected by the disparities and inequities affecting some national health systems. Opening opportunities for higher education and participation in research for health could help tackle some of the inequities affecting them.

Another challenge is the missing pathways to reintegrate trainees into the health research workforce on their return to their homeland. Argentina, Brazil, Chile, and Mexico are developing ways to reintegrate recent graduates who have received scholarships, but most LAC countries do not have a clear and efficient reintegration pathway.

A solution yet to be tested could be the introduction of re-entry grants for returning graduates and researchers and the signing of fair paid licences with service contracts on return that guarantee reintegration with a job and pay scale commensurate with the new skills. This effort will require commitment from and implementation by national research funding agencies, with some support from national and/or international organisations, major donor and/or funding agencies, or private foundations.

## Bridging gaps in governing health research

The challenges to health research governance faced by LAC countries are not unique and are shared by other regions such as South Asia and Sub-Saharan Africa.^14^ ^16^ These include insufficient funding, misalignment of funding with national research priorities, lack of optimal coordination among stakeholders, and limited public health research capacity.^15^

Other major challenges and barriers that persistently affect health research governance in LAC countries include difficulties maintaining up-to-date information on their national health research systems and developments. Several key issues to tackle include the need for continuity, given staff rotation; providing dedicated resources and structured processes; regular monitoring and evaluation of progress made; and incorporating new information and communication technologies to capture information systematically and in real time.

Adopting a “research for health” framework, as PAHO’s policy on research for health does, means dealing with social determinants, transitioning towards a model where different sectors and fields of knowledge, including the health sector, contribute to solutions and provide expertise for meeting health needs and challenges. In LAC countries, this will mean adopting new approaches, going beyond traditional work with peers, and engaging with other sectors to strengthen health research in a sustainable way.^2^

The United Nations, Agenda 2030, sustainable development goals that all 193 member states signed in 2015 further underscore the need for cross-sector collaboration to improve health. The sustainable development goals are strongly aligned with PAHO’s policy on research for health and provide further impetus for international collaboration and support by encouraging reporting on important indicators.

In line with sustainable development goals 3 and 17, a new proposal by the Council on Health Research Development, the research fairness initiative, aims to create a reporting system that encourages governments and other stakeholders to provide data on partnerships in research and innovation.^17^ This could create opportunities for gathering better quality information and relevant data aimed at enhancing research for health governance worldwide.

In January 2017, WHO launched its global observatory on health research and development. For its evaluation framework, the observatory follows the mandate offered in WHO’s strategy on research for health, thereby providing a centralised and comprehensive source of information and analyses on global health research and development.^18^ It shows potential for guiding member states’ efforts to strengthen health research governance. The observatory seeks to collect valuable, up-to-date data from all WHO member states, including LAC countries, on national funding earmarked for health research; bridge persistent gaps in workforce development; and consolidate, monitor, and analyse relevant information on health research and development needs in developing countries, thereby guiding coordinated action. The observatory could serve as a key instrument for policy makers, research sponsors, and others to identify health research and development priorities based on public health needs and then link their indicators to the sustainable development goals.

## Conclusions

Many LAC countries have made substantial efforts to prioritise research for health and enhance research governance, establishing clear policies and programmes. However, LAC countries continue to face challenges and considerable inequities in their capacity to use and produce essential research for health; a number of countries are still searching for specific ways to develop and implement their policies. LAC countries need to harmonise their country level research governance. Pressing demands include building more human capital and adequate research infrastructure to tackle priority public health needs and developing better coordination among all relevant stakeholders—government ministries, national councils of science and technology and innovation, and major universities and research centres. Meeting these demands requires financial support to achieve the objectives set down in national strategies and agendas for health research.

International agencies and other partners could assist by providing financial and technical resources. However, it is essential that LAC countries retain their autonomy in terms of the decision making process. This includes having a say in establishing priorities and financing mechanisms, as well as the means to advance research capacities and outcomes in their countries.

**Key messages**

- Nearly 10 years since PAHO implemented its policy on research for health, countries in Latin America and the Caribbean have progressed, but improvements are still needed in health research governance.
- Greater coordination among all government entities overseeing health research quality is needed in countries across the region.
- The lack of adequate funding aligned with national research priorities remains a limiting factor for many national health research systems.
- Substantial gaps in research skills and capacity remain across the region.
- International organisations and other partners could have an important role in strengthening national health research governance. However, it remains crucial for LAC countries to retain their autonomy in terms of the decision making process, including financing mechanisms.

## Notes

Provenance and peer review: Commissioned; externally peer reviewed.

This article is part of a series proposed by PAHO and commissioned by *The BMJ*, which peer reviewed, edited, and made the decision to publish the article with no involvement from PAHO. Open access fees for the series are funded by PAHO.
